# Evaluation of levosimendan as treatment option in a large case-series of preterm infants with cardiac dysfunction and pulmonary hypertension

**DOI:** 10.1007/s00431-023-04971-9

**Published:** 2023-04-27

**Authors:** Lukas Schroeder, Stanley Holcher, Judith Leyens, Annegret Geipel, Brigitte Strizek, Till Dresbach, Andreas Mueller, Florian Kipfmueller

**Affiliations:** 1 Department of Neonatology and Pediatric Intensive Care Medicine, University Children’s Hospital Bonn, Venusberg-Campus 1, D-53127 Bonn, Germany; 2 Department of Obstetrics and Prenatal Medicine, University Hospital Bonn, Bonn, Germany

**Keywords:** Case-series, Levosimendan, Preterms, Cardiac dysfunction, Pulmonary hypertension

## Abstract

Levosimendan as a calcium-sensitizer is a promising innovative therapeutical option for the treatment of severe cardiac dysfunction (CD) and pulmonary hypertension (PH) in preterm infants, but no data are available analyzing levosimendan in cohorts of preterm infants. The design/setting of the evaluation is in a large case-series of preterm infants with CD and PH. Data of all preterm infants (gestational age (GA) < 37 weeks) with levosimendan treatment and CD and/or PH in the echocardiographic assessment between 01/2018 and 06/2021 were screened for analysis. The primary clinical endpoint was defined as echocardiographic response to levosimendan. Preterm infants (105) were finally enrolled for further analysis. The preterm infants (48%) were classified as extremely low GA newborns (ELGANs, < 28 weeks of GA) and 73% as very low birth weight infants (< 1500 g, VLBW). The primary endpoint was reached in 71%, without difference regarding GA or BW. The incidence of moderate or severe PH decreased from baseline to follow-up (24 h) in about 30%, with a significant decrease in the responder group (*p* < 0.001). The incidence of left ventricular dysfunction and bi-ventricular dysfunction decreased significantly from baseline to follow-up (24 h) in the responder-group (*p* = 0.007, and *p* < 0.001, respectively). The arterial lactate level decreased significantly from baseline (4.7 mmol/l) to 12 h (3.6 mmol/l, *p* < 0.05), and 24 h (3.1 mmol/l, *p* < 0.01).

*  Conclusion*: Levosimendan treatment is associated with an improvement of both CD and PH in preterm infants, with a stabilization of the mean arterial pressure during the treatment and a significant decrease of arterial lactate levels. Future prospective trials are highly warranted.

**Table:** 

**What is Known:**
• *Levosimendan as a calcium-sensitizer and inodilator is known to improve the low cardiac output syndrome (LCOS), and improves ventricular dysfunction, and PH, both in pediatric as well as in adult populations. Data related to critically ill neonates without major cardiac surgery and preterm infants are not available.*
**What is New:**
• *This study evaluated the effect of levosimendan on hemodynamics, clinical scores, echocardiographic severity parameters, and arterial lactate levels in a case-series of 105 preterm infants for the first time. Levosimendan treatment in preterm infants is associated with a rapid improvement of CD and PH, an increase of the mean arterial pressure, and a significant decrease in arterial lactate levels, as surrogate marker for a LCOS.*
• *How this study might affect research, practice, or policy. As no data are available regarding the use of levosimendan in this population, our results hopefully animate the research community to conduct future prospective trails analyzing levosimendan in randomized controlled trials (RCT) and observational control studies. Additionally, our results potentially motivate clinicians to introduce levosimendan as second second-line therapy in cases of severe CD and PH in preterm infants without improvement using standard treatment strategies.*

## Introduction

Today’s challenges in the treatment of critically ill preterm infants are mainly raised by extremely low gestation age newborns (ELGANs), very low birth weight (VLBW), immaturity of lungs, and the acute respiratory distress syndrome (RDS). Putting the neonatal heart in focus, major targets of intensive care treatment are the treatment of an early (< 28 days of life) or late (> 28 days of life) pulmonary hypertension (PH) as well as a cardiac dysfunction (CD) in preterm infants.

Known risk factors for PH or CD in preterm infants are to be found in prenatal and postnatal factors. Prenatal risk factors or conditions putting preterm infants at risk for CD are intrauterine growth restriction (IUGR), discordant twin pregnancies with known complications such as the twin-to-twin transfusion syndrome (TTTS) or selective IUGR (sIUGR), preeclampsia, intrauterine transfusions, or an amniotic infection syndrome [7, 18, 26, 28, 29]. Focusing on PH, major underlying risk factors in VLBW infants comprise RDS, intrauterine conditions with fetal hypoxia (IUGR, TTTS, preeclampsia), and lung disorders leading to lung hypoplasia or vascular remodeling [2, 8, 19].

A new promising drug for the treatment of CD and PH is the inodilator levosimendan. Levosimendan is a calcium-sensitizing drug with positive inotropic, lusitropic, and vasodilating effects, and a lot of research was done in recent years in adults and infants with CD or chronic heart failure. Levosimendan has manifold pharmacological effects: it improves myocardial contractility by increasing the affinity of myocardial troponin C to calcium, induces vasodilation in the smooth muscle cells of the vasculature by opening ATP-dependent potassium channels, and a selective PDE-3 inhibition was described, similar to the effect of PDE-inhibitors such as milrinone [5, 15, 17, 24]. Levosimendan improves both right and left ventricular contractility and is a candidate drug for PH treatment [3, 6, 13].

There are no data analyzing the use of levosimendan in larger cohorts or case-series of preterm infants, apart from case reports [10]. Our study aimed to analyze levosimendan as treatment option for CD and PH in critically ill preterm infants.

## Material and methods

### Patient information

**Fig. 1:**
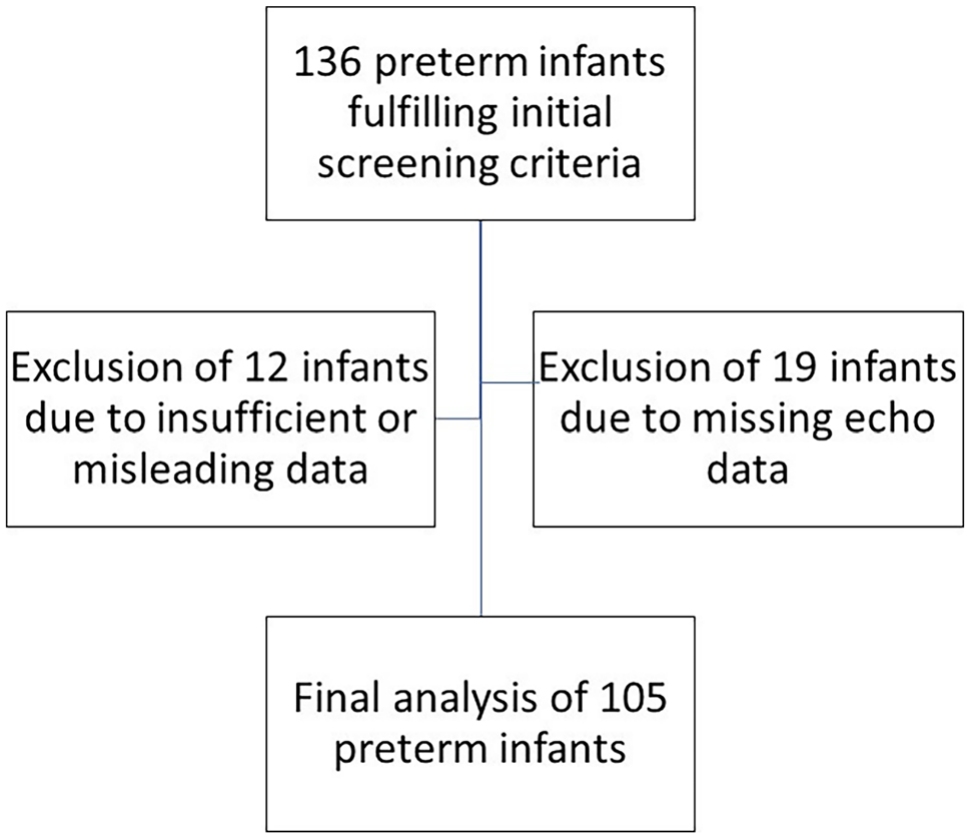
Flow-chart of patient inclusion for the final cohort

Preterm infants (< 37 weeks of GA) treated at the neonatal intensive care unit (NICU) of the University Children’s Hospital of Bonn, Germany, during the study period of 01/2018–06/2021 were retrospectively screened for inclusion in the case-series. Inclusion criteria include documented use of levosimendan, echocardiographic diagnosis of CD, or PH at baseline and available echocardiographic data at baseline and 24 h after onset of levosimendan administration. Exclusion criteria include congenital heart defect with need for operative correction, severe syndromic disorder or chromosomal anomalies, palliative care after birth, and infants with a congenital diaphragmatic hernia (CDH), as levosimendan treatment was already evaluated recently in a subgroup of preterm and term infants with CDH [20]. Of 136 preterm infants (< 37 weeks of GA) detected in the preliminary screening, 105 infants were included in the final analysis, after exclusion of 12 infants due to incorrect data and 19 infants due to missing echo data (see Fig. 1). Baseline characteristics of the study population are displayed in Table 1. The preterm infants (48%) were classified as ELGANs (< 28 weeks of GA) and 73% as very low birth weight (VLBW) infants (< 1500 g).

**Table 1:** Demographic and treatment data

***Variables***	**Overall cohort** ***n*** **= 105**	**Responder** ***n* = 75**	**Non-responder** ***n*** **= 30**	***p*-level**
*Gestational age, w*	28.5 (25.5/32.4)	27.9 (25/32)	29.5 (26/32)	0.382
*Female sex, n (%)*	40 (38)	29 (39)	11 (37)	0.99
*Birth weight, kg*	0.98 (0.7/1.8)	0.9 (0.7/1.8)	0.98 (0.6/1.6)	0.771
*pH umbilical artery*	7.35 (7.3/7.4)	7.35 (7.3/7.4)	7.38 (7.3/7.4)	0.348
*Lowest FiO*_*2*_* in the first 24 h*	0.43 (0.3/0.9)	0.42 (0.3/0.8)	0.55 (0.3/1.0)	0.602
*Apgar 5*	8 (7/9)	8 (7/9)	7 (6/9)	0.362
*Apgar 10*	9 (8/9)	9 (8/9)	9 (7/10)	0.796
*CRIP-score*	8 (3/11)	8 (3/11)	9 (3/14)	0.537
*Primary diagnosis, n (%)* * a) TTTS or sIUGR, n (%)* * b) Fetal hydrops, n (%)* * c) IUGR, n (%)* * d) AIS, n (%)* * e) Congenital lung disorder* * f) Syndromic disorder* * g) Minor malformations*	16 (15) 6 (6) 6 (6) 23 (22) 8 (8) 9 (9) 6 (6)	11 (15) 6 (8) 6 (8) 14 (19) 7 (9) 4 (5) 5 (7)	5 (17) 0 0 9 (30) 1 (3) 5 (17) 1 (3)	0.771 0.179 0.179 0.295 0.435 0.115 0.671
*Complications after birth*				
*Respiratory distress syndrome, grade 3 or 4*	52 (50)	37 (49)	15 (50)	0.703
*Bronchopulmonary dysplasia*	23 (22)	16 (23)	7 (26)	0.791
*Intraventricular hemorrhage, grade 2 or 3*	26 (25)	18 (24)	8 (27)	0.075
*Necrotizing enterocolitis*	7 (7)	5 (7)	2 (7)	0.99
*Sepsis*	36 (34)	28 (38)	8 (27)	0.364
*Interventional or operative PDA closure*	4 (4)	3 (4)	1 (3)	0.99
*Mechanical ventilation, d*	6 (3/12)	6 (3/11)	6 (1/22)	0.956
*Oxygen supplementation, d*	11 (4/54)	11 (5/57)	7 (2/28)	0.304
*In-hospital mortality, n (%)*	26 (25)	16 (21)	10 (33)	0.218

Data are demonstrated as absolute number with percentage or as median values with IQR. Infants with an improvement in right ventricular, left ventricular, or biventricular dysfunction and/ or in PH-severity (min. 1 grade) were defined as responder to levosimendan. A p-value of <0.05 was considered as statistically significant

*AIS* amniotic infection syndrome, *CRIP* clinical risk index for babies, *d* days, *IUGR* selective intrauterine growth retardation, *n* number, *PDA* persistent ductus arteriosus, *TTTS* twin-to-twin-transfusion syndrome, *sIUGR* selective intrauterine growth retardation, *w* week

### Patient’s consent and ethical approval

The study was conducted according to the guidelines of the Declaration of Helsinki and approved by the Institutional Review of the Medical Center of the University of Bonn (local running number 476/22). Informed consent of participants or their parent/ legal guardian was waived due to the retrospective design of the study, as a decision of the local Ethical Committee of the Medical Center of the University of Bonn (running number 476/22).

### Clinical findings

#### Diagnosis and treatment of CD/PH

Diagnosis of CD was based on clinical findings and echocardiographic assessment. A low cardiac output syndrome (LCOS) and CD was suspected in the presence of sinus tachycardia (> 180 bpm), arterial hypotension, low urine output, prolonged capillary refill time, and elevated lactate. Echocardiographic assessment and verification of CD was performed by the attending physician with experience in neonatal echocardiography. The echocardiographic assessment is described below in more detail. When CD was apparent, dobutamine or milrinone were used as first-line inotropes for improvement of ventricular function. In case of arterial hypotension and need for increased afterload, vasopressors (norepinephrine and vasopressin) were added to the inotropic therapy. In infants with missing improvement with the standard drug therapy and severe CD, levosimendan was implemented as a second-line therapy due to the decision of the attending senior physician.

Likewise, the diagnosis of PH consisted of clinical signs of PH and the echocardiographic assessment. Possible clinical signs of PH and oxygen impairment were FiO_2_ > 0.4, pre- and post-ductal SpO_2_-difference > 5%, paO_2_ < 60 mmHg despite oxygen therapy and ventilation support, low mean arterial pressure (MAP), and sinus tachycardia. The echocardiographic PH assessment is described more detailed below. For the reduction of the pulmonary vascular resistance, iNO was used as primary drug therapy when PH was present. In preterm infants with moderate to severe PH, intravenous continuous sildenafil was added as a second-line therapy, followed by bosentan as third-line therapy.

### Diagnostic assessment

#### Echocardiographic measurements

For echocardiographic measurements, a Philips CX50 Compact Extreme Ultrasound system with a S12-4 sector array transducer (Philips Healthcare, Best, the Netherlands) was used. All available echocardiographic data at baseline (prior to start of levosimendan administration) and at follow-up (24 h after onset of levosimendan administration) were retrospectively evaluated offline for analysis independently by two experienced neonatal echocardiographers blinded for the course of the respective infant. Ventricular dysfunction was defined as (a) right ventricular dysfunction (RVD), (b) left ventricular dysfunction (LVD), and (c) biventricular dysfunction (BVD) and classified as: present or not present. For the assessment of ventricular dysfunction, a combined approach of quantitative and qualitative measurements was used, based on international guidelines for neonatal echocardiography [12, 14, 23]. In all patients, stored loops (3–5 s) visualizing the ventricular function (both 4-chamber view and parasternal long-axis) were interpreted via eyeballing assessment [23, 25]. Additionally, fractional shortening (FS; normal: 26–45%, abnormal ≤ 25%) or ejection fraction (EF; normal ≥ 55%, abnormal < 55%) were analyzed when available. The end-diastolic right ventricular to left ventricular (RV/LV) ratio was calculated in a standard four chamber view directly distal to the tricuspid and mitral annulus as a horizontal line from the endocardium of the RV and LV free wall to the endocardium of the interventricular septum. RV dysfunction was assumed when RV/LV-ratio was > 1.0 [23]. Tricuspid and mitral valve regurgitation was further analyzed and graded as I°, II°, or III°. PH was graded as mild, moderate, or severe, using the following echocardiographic parameters: (a) ductus arteriosus (DA) flow pattern, (b) intraventricular septum (IVS) position, and (c) tricuspid valve regurgitation (TVR). Mild PH was diagnosed when DA shunt-flow was left-to-right, IVS was flattened, and TVR was I–II°. Moderate PH was diagnosed when DA shunt-flow was alternating (left-to-right/right-to-left), IVS was flattened, and TRV was II–III°. Severe PH was diagnosed when DA shunt-flow was right-to-left, IVS was D-shaped (towards left ventricular cavity), and TRV was III°.

### Assessment of monitoring data

The following hemodynamic parameters were documented at baseline and at follow-up (3, 6, 9,12, 24, and 48 h after onset of levosimendan drug infusion): systolic and diastolic blood pressure, MAP, heart rate, pre- and post-ductal peripheral oxygen saturation (SpO_2_), and fraction of inspired oxygen (FiO_2_). Arterial blood gas measurements with pH, arterial oxygen partial pressure (paO2), arterial carbon dioxide partial pressure (paCO2), and arterial lactate were documented when available at baseline, and at follow-up (3, 6, 9, 12, 24, and 48 h). For stratification of the oxygenation impairment and for infants with mechanical ventilation (MV), the Oxygenation Saturation Index (OSI; $\frac{\mathrm{FiO}2\mathrm{xMAPx}100}{\mathrm{SpO}2}$) was calculated at baseline and follow-up (12, 24, and 48 h), for infants without MV the Saturation Oxygenation Pressure Index (SOPI; $\frac{\mathrm{CPAP pressure or PEEP x FiO}2}{\mathrm{SpO}2}$) [22] was calculated at the respective timepoints. The vasoactive-inotropic score (VIS) was calculated at the same timepoints according to the formula described elsewhere for estimation of cardiovascular drug support [4].

### Statistical analysis and outcome measures

Infants were divided into subgroups according to the primary outcome: response to levosimendan therapy (responder vs. non-responder). A response to levosimendan treatment was defined as echocardiographic improvement of RVD, LVD, or BVD after 24 h, and/or decrease of PH severity (≥ 1 grade) after 24 h. The following parameters were defined as secondary endpoints or outcome measures: decrease in arterial lactate ≥ 20% after 24 h of levosimendan administration, duration of MV, days of oxygen supply, VIS at 24 h and 48 h, and in-hospital mortality.

For data analysis, SPSS version 27 (IBM Corp., Armonk, NY) was used. Continuous variables were described using median and interquartile range (IQR), and categorical variables were summarized as absolute number (*n*) with percentage. For comparison of continuous and non-normally distributed variables, a Wilcoxon-test or Mann–Whitney *U* test was performed to compare continuous variables between timepoints and subgroups (responder vs non-responder), as appropriate. For categorical variables, the Pearson’s chi^2^ test and Fisher’s exact test were applied, as appropriate. Correlations between variables were evaluated by Spearman correlation coefficients. A *p*-value of < 0.05 was considered significant.

## Results of the therapeutic intervention

### Levosimendan treatment data and oxygenation scores

The primary endpoint (response to levosimendan) was reached in 71%, without difference between VLBW infants/ ELGANs (75%, and 66%) and non-VLBW infants/non-ELGANs (70%, and 78%). The summary of the treatment data is displayed in Table 2. Levosimendan was administered with a continuous infusion over 24 h with a dose of 0.2 µg/kg/min. Only in three preterm infants (3%), a bolus was administered at the start of the infusion (12 µg/kg over 10 min), and in most of the infants (97%), a bolus infusion was waived to avoid an arterial hypotension. In 6 (6%) infants, levosimendan was administered for a second time with a minimum treatment interval of 7 days. No difference was found regarding the concomitant vasoactive treatment and allocation to subgroups (see Table 2).

**Table 2:** Levosimendan treatment data and concomitant cardiac drugs

***Variables***	**Overall cohort**	**Responder**	**Non-Responder**	***p*-level**
	***n* = 105**	***n*** **= 75**	***n*** **= 30**	
***Levosimendan treatment data***				
Start of i.v. levo, DOL	2 (1/3)	2 (1/3)	2 (2/3)	0.484
Levo Bolus at start of infusion (12 µg/kg, 10 min)	3 (3)	2 (3)	1 (3)	0.99
***Concomitant treatment data***				
iNO treatment at start of levo therapy, n (%)	65 (62)	45 (60)	20 (67)	0.657
Dobutamine dose at start of levo treatment, µg/kg/min	7 (5/10)	6 (5/10)	9 (5/10)	0.738
Dobutamine dose at 24 h of levo treatment, µg/kg/min	8 (5/10)	8 (5/10)	7 (5/11)	0.79
Milrinone dose at start of levo treatment, µg/kg/min	0.7 (0.5/0.7)	0.7 (0.5/0.7)	0.7 (0.5/0.7)	0.929
Milrinone dose at 24 h of levo treatment, µg/kg/min	0.7 (0.7/0.7)	0.7 (0.7/0.7)	0.7 (0.7/0.7)	0.952
Norepinephrine dose at start of levo treatment, µg/kg/min	0.25 (0.1/0.5)	0.25 (0.1/0.5)	0.3 (0.1/0.5)	0.893
Norepinephrine dose at 24 h of levo treatment, µg/kg/min	0.3 (0.1/0.5)	0.2 (0.1/0.5)	0.3 (0.1/0.5)	0.946
Vasopressin dose at start of levo treatment, mU/kg/min	0.6 (0.2/1.8)	0.5 (0.2/2.4)	0.6 (0.6/1.7)	0.63
Vasopressin dose at 24 h of levo treatment, mU/kg/min	1.2 (0.5/1.8)	1.0 (0.4/1.9)	1.4 (0.6/1.8)	0.789
**Outcome measures**				
Mechanical ventilation at start of levo treatment, n (%)	42 (40)	32 (43)	10 (33)	0.509
Discharge with oxygen supplementation, n (%)	29 (28)	21 (28)	8 (27)	0.99

Data are presented as median with IQR or absolute number with %. A *p*-value < 0.05 was considered as statistically significant

*d* days, *CPAP* continuous positive airway pressure, *DOL* day of life, *h* hours, *iNO* inhaled nitric oxide, *i.v.* intravenous, *levo* levosimendan, *n* number, *NICU* neonatal intensive care unit

**Fig. 2:**
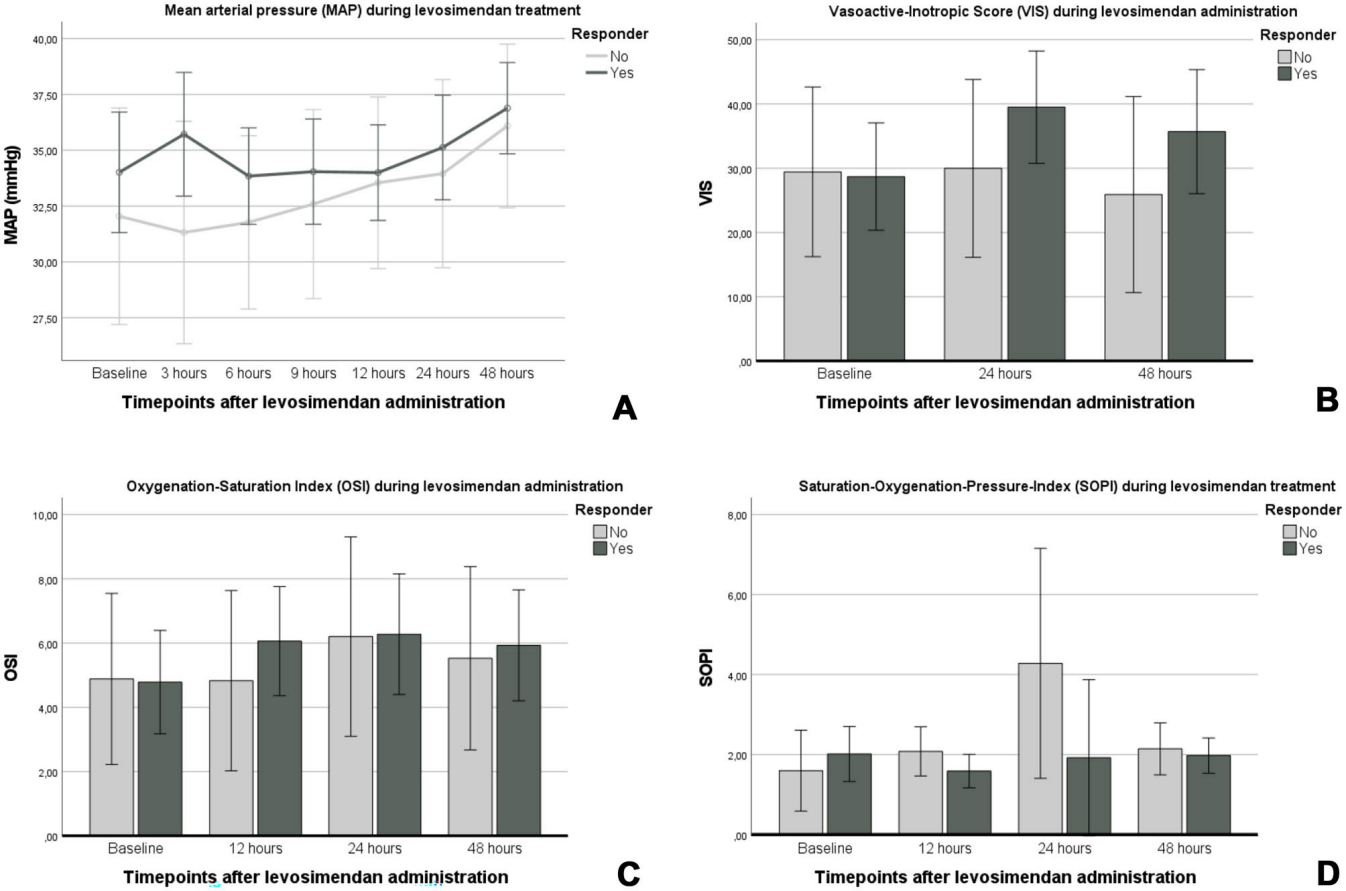
The course of the mean arterial pressure (MAP) during the levosimendan treatment (**A**), the course of the Vasoacitve-Inotropic Score (VIS) during the levosimendan treatment (**B**), the course of the Oxygenation-Saturation Index (OSI), and the Saturation Oxygenation Pressure Index (SOPI) during the levosimendan treatment (**C** and **D**) are displayed for responder and non-responder infants. Data are presented as mean with 95%CI

The MAP remained stable after the onset of levosimendan administration over the first 24 h and increased significantly within 48 h (*p* = 0.05; see Fig. 2a). Regarding the use of vasoactive treatment, no difference was found analyzing the VIS at baseline and at the follow-up timepoints in the overall cohort and between subgroups (see Fig. 2b). The analysis of oxygenation indices (OSI and SOPI) and VIS is displayed in Fig. 2c–d. The arterial lactate decreased significantly from baseline (4.7 mmol/l) to 12 h (3.6 mmol/l, *p* < 0.05), and 24 h (3.1 mmol/l, *p* < 0.01) after the onset of levosimendan administration in the overall cohort. No difference was found between responder and non-responder.

### Echocardiographic assessment

**Fig. 3:**
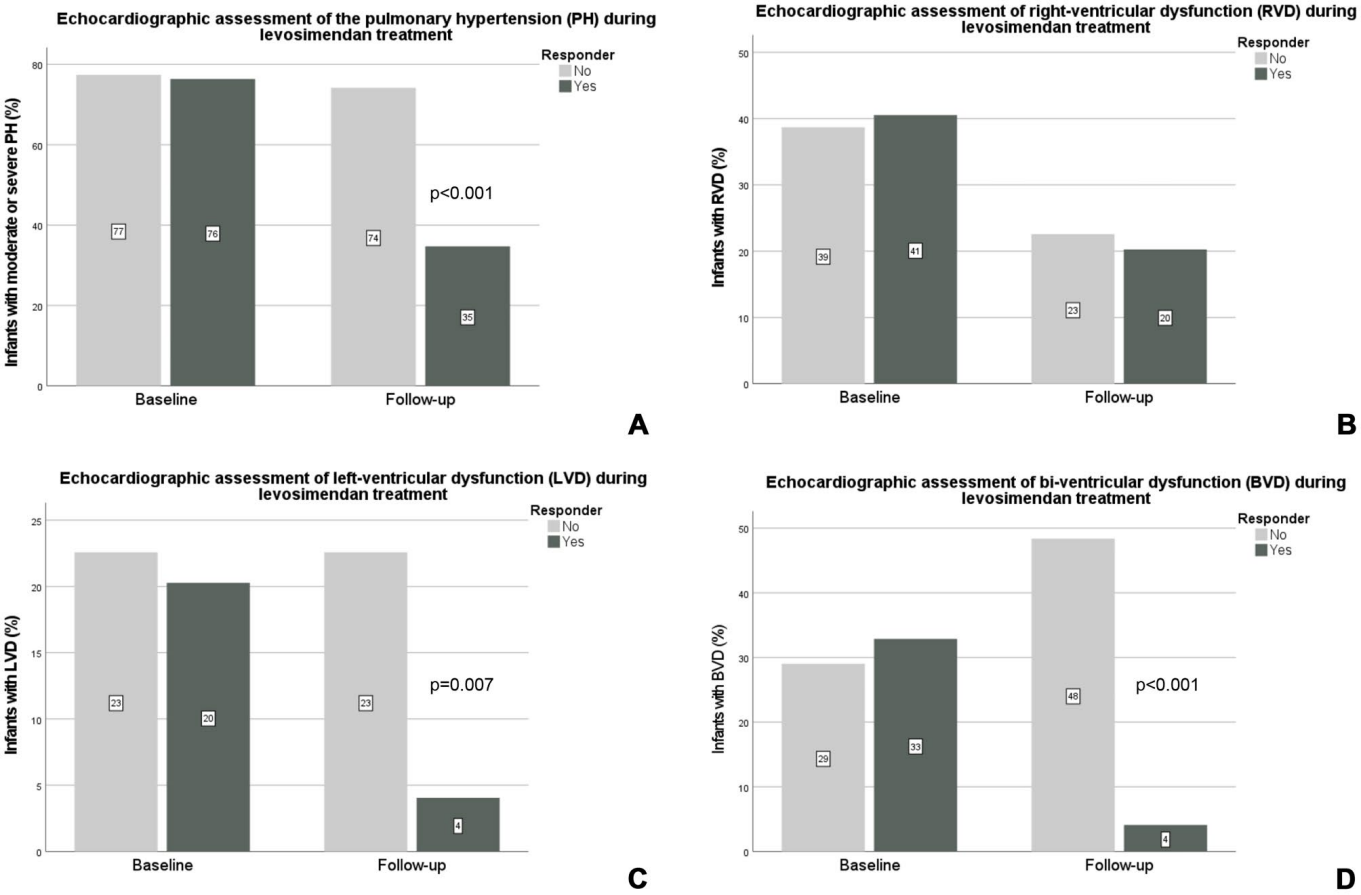
The course of PH (**A**), RVD (**B**), LVD (**C**), and BVD (**D**) during levosimendan treatment at the timepoints baseline and follow-up (24 h) are displayed for responder and non-responder infants. Data are presented as percentage of the respective subgroup. *P*-levels illustrated in figure are related to the difference between subgroups for the related timepoint

**Fig. 4:**
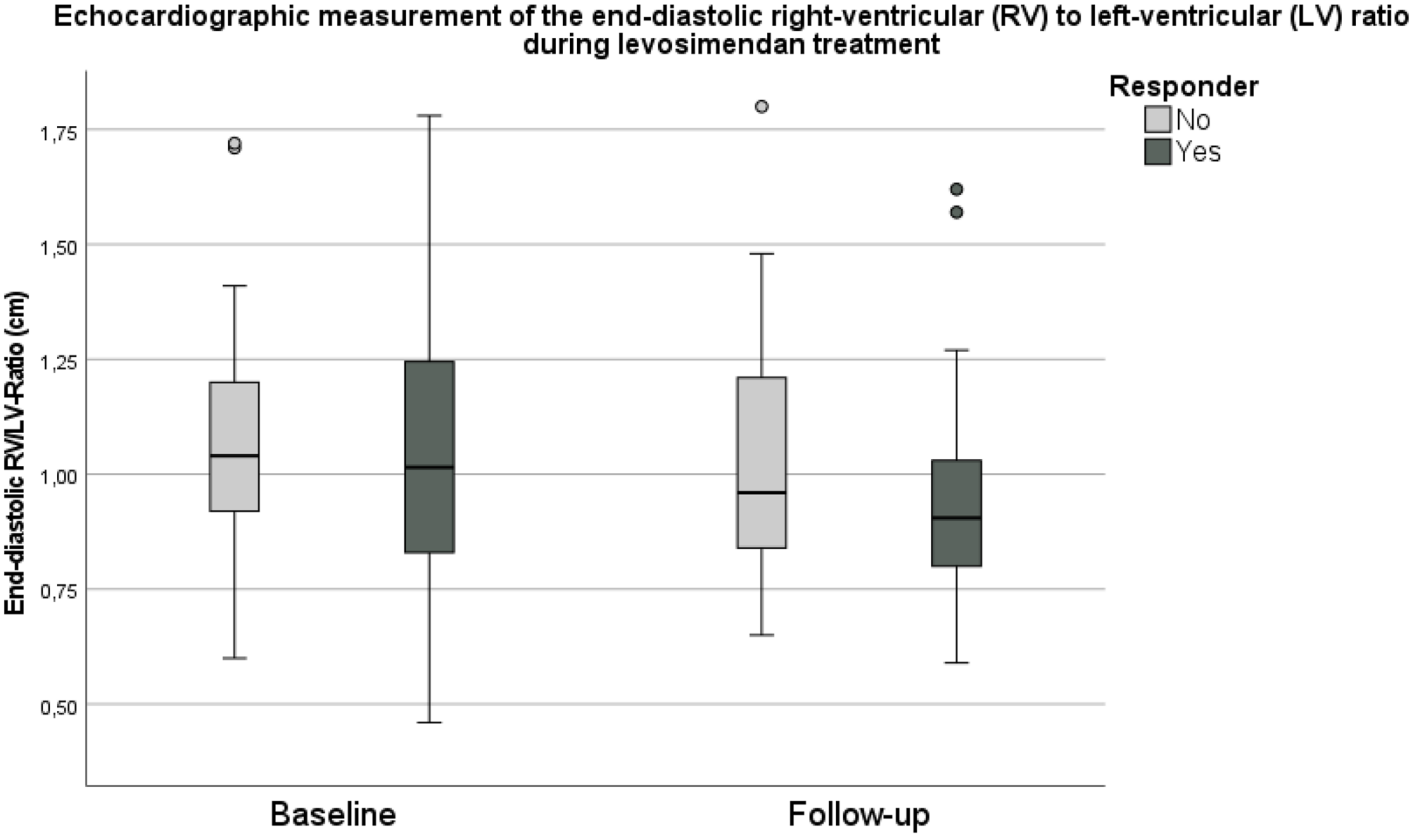
The course of the end-diastolic right ventricular to left ventricular (RV/LV) ratio during the levosimendan treatment is displayed for responder and non-responder infants. Data are presented as boxplots with minimum/maximum, 25/75 quartiles and median

The echocardiographic data are illustrated in Figs. 3a–d and Fig. 4. Moderate or severe PH was present in 77% of the infants. Mild PH was diagnosed in 16% of the infants, and in 8%, no PH was present at baseline. The overall incidence of RVD was 40%, of LVD 21%, and of BVD 31% at baseline. The end-diastolic RV/LV ratio decreased significantly from baseline to follow-up (1.05 vs 0.96, *p* < 0.001), without statistical significance between subgroups (see Fig. 4).

### Safety profile

During the observation period (from baseline to follow-up, max. 48 h), we did not identify a drug-related severe arterial hypotension after starting the levosimendan infusion in the electronic patient’s charts and vital parameter documentation. A severe arterial hypotension was defined as a MAP 5 mmHg < weeks of GA for > 10 min. Additionally, there were no documented cardiac arrhythmias in the retrospective chart review during levosimendan treatment.

## Discussion

The major findings of the present case-series are as follows: over two-thirds of the preterm infants with CD and PH responded to levosimendan treatment. The use of levosimendan is associated with an improvement of both LVD and BVD as well as PH. The response rate to levosimendan is similar in both VLBW infants/ ELGANs, and preterm infants > 28 weeks of GA and with a BW > 1500 g. During levosimendan treatment, the MAP increased significantly from baseline to follow-up (48 h). Additionally, the arterial lactate as surrogate parameter of LCOS decreased significantly after the onset of levosimendan treatment in the first 24 h. However, response to levosimendan was not associated with a reduction in in-hospital mortality.

To our knowledge, only a single case report of levosimendan treatment in a preterm infant is available. Lechner et al. described the successful treatment with levosimendan of a LCOS in a preterm infant of 32 weeks of GA at birth with a transposition of the great arteries [10]. The first experiences of levosimendan treatment in infants were published in the context of pediatric cardiac surgery in 2009 [16], and since several more studies have been published, but research data outside the scope of pediatric cardiac surgery remain scarce [26–28]. Meta-analysis of levosimendan treatment in this population concluded that levosimendan led to an improvement of LCOS, overall hemodynamic stabilization, and reduction of arterial lactate levels, but failed to decrease mortality rates, or the incidence of an acute kidney injury in this population [21, 27]. Nevertheless, the authors stated that levosimendan treatment in the post cardiac-surgery neonatal population is safe and relevant drug-related side effects extremely rare. Similarly, we did not observe drug-related arterial hypotension or arrhythmias during levosimendan administration.

Levosimendan treatment is associated with a significant improvement in RVD and LVD, markers of systolic and diastolic ventricular function, and PH, as previously described in adult populations [6, 9, 11]. Our results are in line with these findings, and levosimendan seems to have a prompt and beneficial effect on myocardial contractility and PH in preterm infants, as a hemodynamic stabilization with higher MAP and lower arterial lactate levels could be observed.

No data are available regarding the effect of levosimendan on oxygenation impairment and the improvement of oxygenation indices. Our data reveal that levosimendan has no effect on the oxygenation impairment in preterm infants, as indicated by the course of the OSI and SOPI after onset of levosimendan treatment (see Fig. 2). As levosimendan is known to improve PH severity in both infants and adults [6, 20], levosimendan can have a positive impact on the oxygenation impairment in critically ill patients. Furthermore, research data show that levosimendan can also improve cerebral oxygenation and peripheral tissue oxygenation in newborns [1].

Underlying prenatal and perinatal complications such as IUGR, TTTS, preeclampsia, or PROM elevate the risk of CD and early PH in preterm infants, with the presence of both comorbidities in many preterm infants. In our cohort, 72% of all infants had a diagnosis of CD in combination with PH. The pharmacologic profile of levosimendan with beneficial effects on the right and left ventricle and the potential to decrease the pulmonary as well as the systemic vascular resistance make levosimendan a promising candidate as an inodilating treatment option in preterm infants.

Preterm infants with an acute or chronic TTTS suffer frequently from a severe dilated cardiomyopathy (DCM, donating cotwin) or hypertrophic (± obstructive) cardiomyopathy (HOCM, recipient cotwin) immediately after birth, with the need in part for multiple cardiac drug therapy [28]. In our case-series, 15% of the infants had a diagnosis of TTTS/ sIUGR (see Table 2). Especially in infants with HOCM, there are limited treatment options in the presence of ventricular dysfunction because classic inotropes such as dopamine/dobutamine further increase the myocardial oxygen demand, increase ventricular muscle-mass, and can therefore worsen HOCM and the patients’ outcome. In infants with DCM, the systolic as well as diastolic function is strongly impaired, and a reduction of afterload is warranted. Levosimendan does not increase myocardial oxygen demand, restores ventricular diastolic function, and reduces right and left ventricular afterload [24]. Therefore, levosimendan can expand pharmacologic treatment options for these infants and should be considered as a first-line inotropic agent. The beneficial effects and its safety profile regarding drug-related side effects make levosimendan a promising drug for cardiac therapy in neonatal population. We hypothesize that levosimendan can be used without preoccupation of arterial hypotension in preterm infants and neonates, but a bolus infusion should be avoided [20].

### Limitations

Retrospective analysis bears the risk of overestimation or underestimation of statistical effects. A comparator cohort is missing, and a prospective randomized trial analyzing the effects of levosimendan as treatment option for CD and PH in preterm infants is highly warranted. Furthermore, the interpretation of the echocardiographic data is at risk for bias because echocardiographic assessment is to some extent operator-dependent, subjective, and based on qualitative grading (eyeball-assessment). By the inclusion of two experienced neonatal echocardiographers for the interpretation of the offline echocardiographic measurements, we tried to reduce potential subjective interpretation.

## Conclusion

Levosimendan treatment is associated with an improvement of both CD and PH in preterm infants, with similar results when adjusting to GA and BW. Furthermore, levosimendan treatment led to a stabilization of the MAP and a significant decrease of arterial lactate levels. The retrospective results need to be interpreted carefully, and future prospective trials are needed.

## Data Availability

The data that support the findings of this study are available on request from the corresponding author. The data are not publicly available due to privacy or ethical restrictions.
